# Effects of online cognitive behavioral therapy on depression, negative automatic thoughts, and quality of life in Thai university students during the COVID-19 lockdown in 2021: a quasi-experimental study

**DOI:** 10.3389/fpsyt.2025.1502406

**Published:** 2025-05-19

**Authors:** Jirada Prasartpornsirichoke, Nuttorn Pityaratstian, Chayanit Poolvoralaks, Nusaba Sermruttanawisith, Kornsiri Polpakdee, Kwunkao Pholphet, Napakkawat Buathong

**Affiliations:** ^1^ Department of Psychiatry, Faculty of Medicine, Chulalongkorn University, Bangkok, Thailand; ^2^ Faculty of Psychology, University College London, London, United Kingdom; ^3^ Department of Psychiatry, King Chulalongkorn Memorial Hospital, Thai Red Cross, Bangkok, Thailand; ^4^ Department of Family Medicine and Preventive Medicine, Faculty of Medicine, Prince of Songkla University, Songkla, Thailand

**Keywords:** online cognitive behavioral therapy, depression, negative automatic thoughts, quality of life, Thai university students, COVID-19 pandemic, social distancing, lockdown measure

## Abstract

**Background:**

The COVID-19 pandemic significantly affected university students’ mental health, family socioeconomic conditions, and social relationships, contributing to increased depression. In Thailand, strict lockdowns and social distancing limited access to in-person psychotherapy.

**Aim:**

The aim of this quasi-experimental single-group study was to investigate the impact of online cognitive behavioral therapy (online CBT) on depressive symptoms, negative automatic thoughts, and quality of life in Thai university students with moderate to severe depression during the 2021 COVID-19 lockdown.

**Methods:**

Seventy Thai university students aged 18–25 participated. Self-reported outcomes were assessed using the Beck Depression Inventory (BDI-IA), the Revised Automatic Thoughts Questionnaire (ATQ-RP), and the WHO Quality of Life-BREF (WHOQOL-BREF-THAI). A mixed-effects model was used to analyze the impact of online CBT at a significance level of 0.05.

**Results:**

Only 33 successfully finished all 12 online CBT sessions and the follow-up assessment. This indicates a dropout rate of 52.9%. The findings revealed a negative linear correlation between the number of CBT sessions and both depressed symptoms and negative automatic thoughts. On the other hand, we observed a positive linear association with quality of life. At the beginning of the study, the mean scores for depressive symptoms significantly decreased from 27.84 (95% CI: 25.56-30.13) to 11.47 (95% CI: 8.54-14.40) at the twelfth session and further declined to 9.43 (95% CI: 6.41-12.46) during the follow-up period. Similarly, the mean scores of negative automatic thoughts decreased from 20.81 (95% CI: 19.08-22.55) to 10.46 (95% CI: 7.96-12.96) and 9.38 (95% CI: 6.77-11.99) respectively. On the other hand, the mean scores for quality of life increased from 69.73 (95% CI: 66.88-72.58) to 85.37 (95% CI: 80.83-89.90) and 85.61(95% CI: 80.84-90.38) consecutively.

**Conclusions:**

This study provides evidence that the implementation of online cognitive behavioral therapy leads to a significant reduction in depression symptoms and negative automatic thoughts, as well as an improvement in the quality of life among Thai university students during the COVID-19 lockdown. The results highlight the benefits and availability of online cognitive behavioral therapy as a mental health intervention during challenging times and in geographically isolated regions.

**Clinical trial registration:**

https://www.thaiclinicaltrials.org/ identifier, TCTR20210310002.

## 1 Introduction

Depressive symptoms, such as difficulties in concentration, sadness, anhedonia, fatigue, and negative self-perception, adversely affect an individual’s quality of life (1) and daily functioning (2). Higher levels of depressive symptoms can lead to significant impairments in various dimensions of life quality. Depression and anxiety are prevalent among university students, negatively impacting their academic achievement and relationship issues (3). A systematic review indicated that the global prevalence of depression among undergraduate students was 30.6%, with ratios ranging from 10% to 85% (4). Demographic and psychological characteristics such as being female, being a first-year undergraduate, having a family history of depression, having sleep difficulties, time management, substance use behaviors, and so on are significant risk factors for depression in university students (3, 5–7). Conversely, academic self-efficacy is associated with a lower risk of depression (8). Addressing depressive symptoms during early adulthood is crucial, as they can lead to long-term consequences, including physical multimorbidity in middle age and reduced future income, ultimately affecting quality of life (9–11). Given the significant impact of depression on young adults, universities should implement comprehensive mental health programs or psychotherapy treatment to support students. Early identification and intervention for those at risk, along with the promotion of a supportive academic environment, can mitigate the adverse effects of depression and improve overall student well-being.

The COVID-19 pandemic created an unprecedented global crisis from 2019 to 2022, significantly impacting various professional activities and the lives of individuals worldwide (12). In Thailand, the government implemented strict nationwide lockdown measures on at least four occasions between 2020 and 2022. The first lockdown occurred in March 2020, and the last occurred in July 2021. The government required non-essential businesses and shops to temporarily close and advised people to stay at home except for necessary outings. This resulted in the loss of many people’s temporary or permanent jobs, as well as their placement on leave (13). Additionally, the closure of schools and universities resulted in the cancellation of examinations and a shift to online education at all levels. The sudden lockdowns and concerns about the COVID-19 pandemic, exacerbated by insufficient information, caused significant stress and mental health problems in the Thai population, leading to a higher prevalence of depression during the pandemic compared to normal times. A study in Northern Thailand reported a depression ratio of 27% (14), which is slightly higher than the pre-pandemic ratio of 21.1% (15). This is consistent with the estimated global prevalence of depression among medical students, which ranges from 7.45% to 48.30% (16).

The COVID-19 pandemic has had a negative impact on the mental health of university students. Studies link the spread of misinformation through social media platforms to higher ratios of depression, anxiety, and psychological problems (17, 18). The implementation of strict lockdown measures and reduced social interactions have further contributed to decreased social support for students, compounding mental health challenges (19). The temporary closure of universities and the transition to online learning platforms also posed a significant impact on students’ academic achievement, critical thinking, mental health, and social lives (20–24). Adapting to virtual learning presented additional challenges for students, requiring them to modify their learning techniques and navigate online discussions for the first time ever (25–28).

Cognitive behavioral therapy (CBT) is a widely used and effective psychotherapy for depression that encompasses a wide range of intervention approaches that include psychoeducation, behavioral activation, and problem solving (29, 30). These therapies can be applied singly or in different combinations. Research has extensively studied CBT and proven its effectiveness in reducing depression symptoms and preventing relapse (31). However, the specific mechanisms underlying its effectiveness remain obscure (32). Cognitive-behavioral models propose that thoughts, beliefs, and behaviors play a crucial role in depression; thus, modifying dysfunctional thoughts and biases in information processing is key to addressing depression (33).

CBT is being delivered using multimedia platforms more frequently in order to improve accessibility in self-help or hybrid formats (34). Over the past decade, the Internet has facilitated a significant rise in the use of treatments such as online cognitive behavioral therapy (online CBT) or internet-based cognitive behavioral therapy (iCBT). Compared to traditional psychotherapy, iCBT is less expensive and helps patients overcome their reluctance to seek treatment (35). Moreover, computer-assisted cognitive behavioral therapy (cCBT) has enormous potential as a scientifically supported approach to overcome the drawbacks of conventional in-person therapy. cCBT effectively addresses various obstacles, including a shortage of therapists, high expenses, limited availability in remote regions, individualized methodologies, anonymous access, and a reluctance to seek assistance. Consequently, treatment becomes more accessible and capable of accommodating a larger number of individuals (36). Computer-based and Internet-based therapies have become popular methods that use technology to deliver therapy through interactive multimedia forms (37). Grist and Cavanagh’s research (2013) has shown that cCBT significantly improves mental well-being by reducing symptoms of sadness and anxiety (38). Moreover, the widespread availability of the Internet in educational institutions makes it an invaluable resource for students. Self-help therapies consist of predefined protocols that do not include any interaction with a therapist. On the other hand, hybrid interventions mix in-person sessions with multimedia features (39).

Traditional CBT typically involves face-to-face sessions with a therapist, where individuals work together to identify, challenge, and evaluate thoughts that contribute to depressive moods (33). Online CBT uses information technology to provide psychological treatment. The format of online CBT varies, but it generally involves structured self-help through online written materials and/or audio/video files, guided by a therapist (40–42), or individual consultations with a therapist. The flexibility of online CBT addresses the limitations of traditional CBT, such as scheduling constraints, geographical distance, and high costs. With online CBT, individuals can access therapy sessions at their preferred time and location. Growing evidence supports the clinical effectiveness of online CBT for depression compared to treatment as usual (42, 43). Online CBT is convenient and compatible with participants’ daily routines, accessed from home computers. Online CBT fosters a virtual therapeutic relationship and facilitates the expression of thoughts and emotions (44). Thus far, depression patients who are comfortable with computers, value written communication and reflection, value anonymity, and are receptive to CBT’s active nature have responded positively to CBT (45).

Although the absence of visual cues and immediate responses in online CBT may pose challenges and potentially exacerbate negative thoughts in individuals with depression, online CBT remains a crucial intervention for alleviating stress, anxiety, and depression. This is particularly relevant during periods of social distancing and lockdowns necessitated by the COVID-19 pandemic. By utilizing internet-based platforms, online CBT enables remote therapist-client interactions, allowing mental health professionals to provide psychological support without the need for in-person consultations. Previous studies have demonstrated that online CBT and counseling are as effective as face-to-face therapy in reducing perceived stress, anxiety, and depression among university students experiencing emotional distress (46–49).

The stringent lockdown measures and rigorous enforcement of social distance protocols in Thailand hindered university students with low moods from accessing conventional psychotherapeutic interventions. This quasi-experimental study sought to conduct online cognitive behavioral therapy (online CBT) with Thai university students experiencing moderate to severe depression. The study aimed to examine the impact of online CBT on negative automatic thoughts, depressive symptoms, and quality of life among participants during the COVID-19 pandemic lockdown in 2021.

## 2 Materials and methods

This quasi-experimental study has received approval from the Institutional Review Board of the Chulalongkorn University Faculty of Medicine (IRB no. 069/64 and COA no. 432/2021, no. 0351/2022). It has been registered for a clinical trial in the Thai Clinical Trials Registry (TCTR) under the number TCTR20210310002. This study utilized a single-group design to investigate the effects of online CBT under the condition of lockdown in Thailand during the data collection period from July to November 2021. Participants provided consent in two instances: initially, by actively expressing their interest at the commencement of the recruitment process, demonstrating their willingness to participate in the research project, and subsequently, by signing a written consent form prior to attending the online cognitive behavioral therapy (CBT) sessions. The first online CBT session was conducted in July 2021, and the intervention continued until November 2021.

### 2.1 Recruitment and procedure

The participants in this study were Thai university students who experienced moderate to severe depression. We employed digital posters as a method of engaging university students through online platforms. Students have the option to register for the study by scanning a QR code and responding to screening questions if they suspect they might be experiencing depression and are willing to participate in the research (consent through action). We assessed the presence of depression using screening questionnaires, which were based on the 2Q and PHQ-9 depression questionnaires in Thai. The eligibility criteria encompassed individuals aged 18–25 who passed the 2Q questions, obtained a PHQ-9 score of 9 or more (indicating moderate to severe depression), and possessed a smart mobile phone or another personal communication device with internet access for online cognitive behavioral therapy.

We used the following criteria to exclude participants: prior exposure to any form of cognitive behavioral therapy (CBT), ongoing treatment for major depressive disorder (MDD), and use of antidepressant medication. The significant risk of suicide thoughts and attempts during the online cognitive behavioral therapy (CBT) sessions, along with the repeated refusal to attend online sessions for two consecutive weeks, justified the withdrawal from the trial. Participants who disclose suicidal ideation will receive immediate intervention and support from a CBT therapist. Subsequently, a child and adolescent psychiatrist associated with the research project will evaluate the participant’s condition. In the case of an emergency, the participant will be referred to a hospital where they can receive the necessary treatments.

A total of sixty-six individuals attended the first online cognitive behavioral therapy (CBT) session in July 2021. We implemented a twelve-week online CBT program for depression, gathering data at five intervals: week 0 (baseline, prior to the initial CBT session), week 4, week 8, week 12, and week 13 (follow-up, one week after concluding the 12-session program). All evaluations were administered online via Google Forms. A final sample of 47 participants who completed a minimum of the first four CBT sessions was included for analysis.

### 2.2 Intervention of the study

#### 2.2.1 Online Cognitive Behavioral Therapy

Online cognitive behavioral therapy (online CBT) denotes a form of cognitive-behavioral intervention administered via an online platform, such as Zoom or Google Meet. It facilitates personalized interactions between CBT therapists and participants. This technique closely follows the traditional cognitive behavioral therapy (CBT) framework, which consists of two primary components: the cognitive aspect, addressing maladaptive thoughts, and the behavioral aspect, focusing on maladaptive behaviors that may stem from either maladaptive or adaptive beliefs. The principal techniques for altering maladaptive beliefs include Socratic questioning, the downward arrow technique, and cognitive thought assessment. These techniques specifically seek to challenge and reformulate detrimental or inaccurate beliefs that lead to emotional distress. Behavioral activation and behavioral experimentation are the two predominant techniques for altering maladaptive behaviors. These strategies seek to motivate individuals to participate in activities that foster positive behaviors and investigate innovative methods of action, thereby enhancing their overall well-being and functionality.

The research conducted a twelve-week online CBT program for depression, comprising twelve one-hour sessions. Weekly sessions were arranged. The research project enabled participants to prolong their online CBT sessions by an additional week. The research project will exclude participants who miss two consecutive weeks of online CBT sessions.

The online CBT program consists of twelve sessions that adhere to a well-defined and organized approach. **Table 1** presents a schematic overview of the 12 sessions of the online CBT program. The first session begins with a brief interview to determine the therapy’s objectives and provide self-monitoring tasks. During Sessions 2 and 3, the therapist motivates clients to openly talk about their problems, employing methods to recognize cognitive distortions. Sessions 4 to 10 focus on addressing individual difficulties by applying Socratic questioning and cognitive restructuring techniques to change maladaptive thinking. Additionally, we utilize behavioral activation to address behavioral problems like social withdrawal. Each session includes homework assignments for practicing and applying the skills learned. Sessions 11 and 12 are mostly about reinforcing the process and keeping symptoms from coming back. This is done by looking at progress, problems that are happening now, and ways to spot early warning signs based on past experiences. This approach ensures a thorough therapy for depression.

**Table 1:** A schematic overview of the 12 sessions of the online CBT program.

Session	Intervention	Target
1	Assessment Psychoeducation about CBT and depression	- Evaluation to comprehend and acquire information for the formulation of the problem list (encompassing both cognitive and significant behavioral aspects). - For developing a protocol that aligns with their urgent issues - To comprehend CBT, its mechanisms in addressing issues, and its efficacy in reducing depression.
2	Psychoeducation about the connection between cognition and emotions, and the basic of emotion	- To comprehend the influence of cognitive processes (automatic thoughts, intermediate beliefs, core beliefs) on emotions, including physical reactions and behaviors. - To understand the significance of each emotion and how we can derive cognitive insights from them.
3	Behavioral activation 1	- To determine the beneficial factors that improve mood through specific behaviors, ultimately leading to increased positive emotions.
4	Behavioral activation 2	- To review the activities and behaviors previously established in the schedule from the last session.
5	Unhelpful thinking style 1	- To identify unhelpful thinking patterns that influence automatic thoughts, and to consider whether constructive thinking styles may contribute to the persistence of problems.
6	Unhelpful thinking style 2	- To identify unhelpful thinking patterns that influence automatic thoughts, and to consider whether constructive thinking styles may contribute to the persistence of problems.
7	Cognitive restructuring 1	- To assess cognition by inquiring into the validity, alternatives, and utility of the thought.
8	Cognitive restructuring 2	- To assess cognition by inquiring into the validity, alternatives, and utility of the thought.
9	Maintaining cycle and maintaining factor	- To comprehend the complexities of depression and identify the maintenance cycle and factors that sustain the issue.
10	Functional analysis of behavior	- To understand the persistence and maintenance of certain maladaptive behaviors. Set the behavior target to change.
11	Problem solving	- To address issues that cannot be resolved through cognitive or behavioral techniques.
12	Relapse prevention	- To monitor warning signs of potential depression recurrence. - Evaluation of all skills acquired through role-playing.

Sixty-six CBT therapists were voluntarily recruited for the research program through social media platforms. Participants were matched with therapists on a one-to-one basis based on schedule availability. All therapists were certified as Cognitive Behavioral Therapists through the Diploma Program in Mental Health: Cognitive Behavioral Therapy at the Faculty of Medicine, Chulalongkorn University. Each therapist possessed a minimum of two years of professional experience in therapeutic practice. Prior to conducting online CBT sessions, the research team provided standardized training to all participating therapists.

### 2.3 Measurements of outcomes

#### 2.3.1 Quality of life

We used the World Health Organization Quality of Life-BREF in Thai version (WHOQOL-BREF-THAI) to assess five aspects of quality of life in this study: overall quality of life, physical health, mental health, social relationships, and environment. This self-report questionnaire includes 23 positive items and three negative items (reverse scoring) on a five-point Likert scale (e.g. 1 = not at all, 5 = extremely), with an overall score range between 26 and 130. There are two ways to interpret the questionnaire’s scoring. Higher scores signify an improved quality of life. Secondly, a total score ranging from 26 to 60 indicates poor quality of life, 61 to 95 denotes moderate quality of life, and 96 to 130 represents good quality of life. The original version of the questionnaire has shown good reliability and validity. The Thai version of the questionnaire also has robust reliability and validity, with a Cronbach alpha coefficient of 0.84 and a content validity of 0.65 compared to the WHOQOL-100 questionnaire in Thai.

#### 2.3.2 Depression

This study employed the Thai version of the Beck Depression Inventory IA (BDI-IA) to assess the participants’ depressive symptoms. The BDI-IA consists of 21 items evaluated on a four-point Likert scale, with 15 items pertaining to psychological symptoms and six items regarding physical symptoms. The questionnaire’s scoring can be interpreted in two ways. Scores vary from 0 to 63, with elevated scores reflecting greater severity of depression. Secondly, a total score of 0–13 indicates minimal depression, 14–19 signifies mild depression, 20–28 denotes moderate depression, and 29–63 reflects severe depression. The instrument exhibits robust content validity (0.84), reliability (Cronbach’s alpha coefficient of 0.92), sensitivity (84.6%), and specificity (86.4%).

#### 2.3.3 Negative automatic thoughts

We assessed participants’ negative automatic thoughts over the past week using the revised Thai version of the Automatic Thoughts Questionnaire (ATQ-RP). This questionnaire comprises 30 items with yes-no responses. Scores range from 0 to 30, with higher scores indicating an increase of negative automatic thoughts. It demonstrates good reliability, with a Cronbach’s alpha coefficient of 0.90.

This study comprised five repeated self-reports of QOL, BDI-IA, and ATQ from the participants. The preliminary evaluation occurred the day prior to the inaugural online CBT session (baseline). The second to fourth assessments were conducted during the online CBT intervention, the day before the fourth, eighth, and twelfth sessions. The follow-up assessment was conducted one week after the conclusion of the twelve sessions of online cognitive behavioral therapy.

### 2.4 Statistical Analysis

This research employed STATA 18.0 for dataset analysis. We utilized a mixed-effects linear regression model to examine the impact of online cognitive behavioral therapy (CBT) on depressive symptoms, negative automatic thoughts, and quality of life. The statistical analysis comprised forty-seven Thai university students who participated in a minimum of four out of twelve sessions and underwent outcome assessments at two intervals (baseline and week 4). We utilized descriptive statistics to present participant characteristics and personal information, including mean, standard deviation (SD), and frequency.

Mixed-effects linear regression models addressed intrasubject correlations. The models incorporated a fixed effect for time, regarded as a categorical variable, and sex (female =1) as a covariate to account for potential confounders. We employed Maximum Likelihood Estimation (MLE) for parameter estimation, setting a significance level of 0.05.

## 3 Results

**Figure 1:**
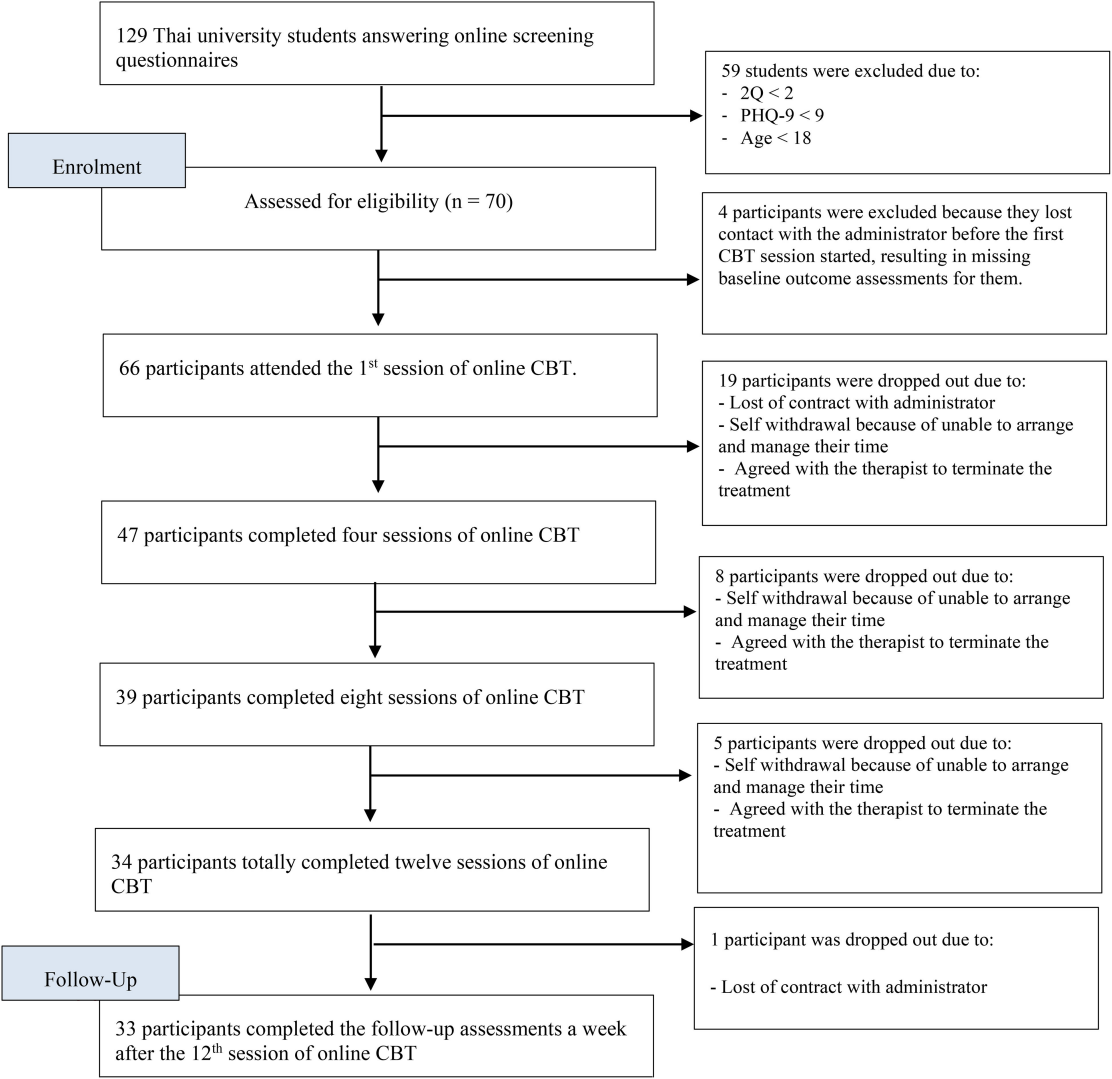
Participant recruitment flow.

This research employed a quasi-experimental design involving a single group to examine the impact of online CBT on depressive symptoms, negative automatic thoughts, and quality of life among Thai university students. Thirty-three of the recruited participants successfully completed all twelve online cognitive behavioral therapy sessions and their follow-up evaluation. The research reported a dropout rate of 52.90%. **Figure 1** depicts the flowchart for participant recruitment. The preliminary assessment conducted one day prior to the first online CBT session revealed that a significant majority of participants were female (78.79%) and had no history of psychiatric issues during childhood (93.94%). The average age was 20.97 years, accompanied by a standard deviation of 1.90 years. **Table 2** describes the characteristics of the participants.

**Table 2:** Demographic of participants.

Factors	At baseline (n=66)		At Week 4 (n=47)	
	Number (%)	Mean (S.D.)	Number (%)	Mean (S.D.)
Sex				
Female	52 (78.79)		39 (82.98)	
Male	14 (21.21)		8 (17.02)	
**Age**		20.97 (1.90)		20.91 (1.79)
Psychiatric problem				
No	62 (93.94)		44 (93.62)	
Yes	4 (6.06)		3 (6.38)	
-Anxiety	2 (3.03)		2 (4.25)	
-ADHD	2 (3.03)		1 (2.13)	

**Table 3** displayed the findings from the mixed-effects linear regression analysis. The provided data contains the least squares means and 95% confidence intervals for the BDI-IA, ATQ, and QOL scores. We conducted the assessments at the baseline (reference group), week 4, week 8, week 12, and follow-up. The results reported in **Table 3** demonstrate that online CBT has a statistically significant effect on the continued decrease of depressive symptoms and negative automatic thoughts, as well as the enhancement of quality of life. The average BDI-IA scores showed a significant decrease from 27.84 at the beginning to 19.23 (95%CI: 16.90, 21.55, p-value <0.001) at week 4, 17.03 (95%CI: 14.43, 19.63, p-value <0.001) at week 8, 11.47 (95%CI: 8.54, 14.40, p-value <0.001) at week 12, and continued to decline during the follow-up assessment (least square mean = 9.43, 95%CI: 6.41, 12.46, p-value <0.001). Also, the least squares mean of ATQ scores went from 20.81 at the start to 16.34 (95%CI: 14.55, 18.14, p-value <0.001) at week 4, 13.70 (95%CI: 11.59, 15.81, p-value <0.001) at week 8, 10.46 (95%CI: 7.96, 12.96, p-value <0.001) at week 12, and it continued to decrease until it reached 9.38 (95%CI: 6.77, 11.99, p-value <0.001) in the follow-up assessment. In contrast, online CBT greatly enhanced the quality of life for Thai university students with moderate to severe depression. The average WHOQOL score steadily climbed from 69.73 at the beginning to 75.33 (95%CI: 72.31, 78.34, p-value < 0.001) at week 4, 78.70 (95%CI: 75.01, 82.39, p-value < 0.001) at week 8 and 85.37 (95%CI: 80.83, 89.90, p-value < 0.001) at week 12. It marginally increased further at the follow-up assessment (mean = 85.61, 95%CI = 80.84, 90.38).

**Table 3:** The effects of online CBT on depressive symptoms, negative automatic thoughts, and quality of life among Thai university students.

Variables	BDI-IA		ATQ		QOL	
	Mean (95%CI)	*p*-value	Mean (95%CI)	*p*-value	Mean (95%CI)	*p*-value
Baseline	27.84 (25.56-30.13)	Ref.	20.81 (19.08-22.55)	Ref.	69.73 (66.88-72.58)	Ref.
Week 4	19.23 (16.90-21.55)	< 0.001	16.34 (14.55-18.14)	< 0.001	75.33 (72.31-78.34)	< 0.001
Week 8	17.03 (14.43-19.63)	< 0.001	13.70 (11.59-15.81)	< 0.001	78.70 (75.01-82.39)	< 0.001
Week 12	11.47 (8.54-14.40)	< 0.001	10.46 (7.96-12.96)	< 0.001	85.37 (80.83-89.90)	< 0.001
Follow-up	9.43 (6.41-12.46)	< 0.001	9.38 (6.77-11.99)	< 0.001	85.61 (80.84-90.38)	< 0.001

Values are presented as least square means and 95% confidence interval (95%CI). p-values represent the results of linear mixed model analysis with outcome as the dependent variables in BDI-IA, ATQ, and QOL. In the model, subjects were random factors. Weeks of assessments was fixed factors and participants’ sex (female =1) was covariate.

## 4 Discussion

This study aimed to examine the effects of an online CBT intervention on depressive symptoms, negative automatic thoughts, and quality of life in Thai university students experiencing moderate to severe depression during the COVID-19 pandemic lockdown in 2021. This study’s findings indicated that online CBT effectively reduces depressive symptoms and negative automatic thoughts, while enhancing the quality of life for Thai university students. Numerous systematic reviews and meta-analyses found that traditional cognitive-behavioral therapy (CBT), internet-based CBT (iCBT), and computer-based CBT (cCBT) are robustly supported and documented effective psychotherapies for alleviating and preventing depression and anxiety in adolescents and young adults (50–56).

According to the analysis of Thai university students receiving online CBT for four-to-twelve consecutive weeks, the result implied that receiving at least four consecutive weeks could significantly reduce the depressive symptoms. Furthermore, the mean depression scores among participants changed from a moderate level to a minimal level of depression when completing twelve sessions of CBT. This suggested that twelve standard sessions of online cognitive-behavioral therapy were appropriate for treating depression. This aligns with the prior study indicating that participants who attended a minimum of 12 sessions of CBT likely perceived it as a learning process and utilized skills for managing depression (57).

The significant decrease in negative automatic thoughts reflects CBT’s core mechanism for challenging and restructuring negative thought patterns. Beck’s cognitive theory (33) posits that negative automatic thoughts are central to depression, and their reduction is a key indicator of therapeutic success. The result was in line with previous study (58) that online CBT effectively addresses these dysfunctional thoughts, which may in turn lead to reduced depressive symptoms.

The improvement in quality-of-life scores suggests that as depressive symptoms and negative thoughts decreased, university students experienced better overall well-being and functioning. This finding is critical because it demonstrates the broader benefits of online CBT beyond symptom reduction, emphasizing its role in improving quality of life and overall mental health. This result was in line with the systematic review that CBT can improve quality of life by having severe depressive symptoms and negative automatic thought as the moderators (59).

The efficacy of online CBT in reducing depression, restructuring negative thoughts, and improving quality of life is highly relevant in the context of modern mental health service delivery. As digital interventions become increasingly popular due to their flexibility, cost-effectiveness, and accessibility, this study suggests that online CBT could be a valuable tool for addressing mental health needs in university settings, especially in low-resource environments such as Thailand.

There were several limitations in interpreting these findings. First, the study design lacked a control group due to the lockdown measures implemented during the COVID-19 pandemic, which limits the ability to draw causal conclusions about the effectiveness of online CBT. Future studies should consider employing a randomized controlled trial (RCT) design to strengthen the evidence for causality.

Secondly, the relatively small sample size and the high dropout rate (52.9%) limit the generalizability of the results. The dropout rate, while common in online interventions, may reflect challenges in maintaining engagement in digital therapy. This could bias the results, as those who completed the study may have had higher motivation or different baseline characteristics than those who dropped out.

The high dropout rate in this study stemmed from several factors. Despite furnishing participants with detailed information regarding the research project and having an administrator confirm appointments one day prior to each online CBT session, attrition remained an issue. The primary cause of dropout was participant withdrawal. A significant number were unable to adhere to the 12-week online CBT program as originally agreed, citing challenges in time allocation. Certain participants perceived that CBT-based psychotherapy was inappropriate for their needs. Some were unwilling to do the designated homework. Some individuals experienced abrupt mood shifts that caused them to withdraw from the program or intentionally rendered themselves inaccessible by disabling their communication devices. The second reason for dropout resulted from an agreement between participants and therapists, as participants’ depressive symptoms had alleviated to a normal level, resulting in a termination of their participation in the program. For future studies, we recommend a shorter online CBT program, such as six or eight sessions, which has demonstrated clinical efficacy and may be more practical for university students.

Thirdly, the study relied on self-reported questionnaires to identify and measure depressive symptoms. Despite their widespread use and validation, these tools’ inherent subjectivity may not fully capture the complexity of participants’ mental health status. Personal bias, mood fluctuations, or social desirability could influence participants’ responses, potentially impacting the accuracy of the results.

Finally, while this study focused on short-term outcomes, it is essential to assess the long-term sustainability of these effects. To determine whether the benefits of online CBT persist over time, future research should incorporate longer follow-up periods.

In conclusion, this study contributes to the accumulating evidence supporting the impact of online cognitive-behavioral therapy (CBT) in alleviating depressive symptoms and negative automatic thoughts, thereby improving the quality of life among university students. The results indicate that online CBT is an effective intervention for addressing mental health issues in young adults, particularly in situations where traditional mental health services are less accessible, highlighting its potential as a scalable and convenient alternative to in-person therapy. A key strength of this study is its investigation of the effects of online CBT on depression, automatic negative thoughts, and quality of life during the COVID-19 pandemic in Thailand, providing valuable insights for future mental health interventions and preparedness strategies in similar public health crises. However, several limitations must be acknowledged, including the lack of a control group, small sample size, high dropout rate, reliance on self-reported outcome measures, and short-term follow-up, all of which may limit the generalizability and robustness of the findings. Future research should address these limitations by incorporating larger samples, randomized controlled trials, objective outcome assessments, and extended follow-up periods. Moreover, decreasing the number of CBT sessions to 6 or 8 to mitigate dropout rates and examining the mechanisms that contribute to the efficacy of online CBT could further augment its implementation and influence in mental health care.

## Data Availability

The raw data supporting the conclusions of this article will be made available by the authors, without undue reservation.
